# In vivo activities of heparan sulfate differentially modified by NDSTs during development

**DOI:** 10.1002/pgr2.17

**Published:** 2024-02-20

**Authors:** Eriko Nakato, Sarah Baker, Akiko Kinoshita-Toyoda, Collin Knudsen, Yi-Si Lu, Masahiko Takemura, Hidenao Toyoda, Hiroshi Nakato

**Affiliations:** 1 Department of Genetics, Cell Biology, and Development, University of Minnesota, Minneapolis, Minnesota, USA; 2 Faculty of Pharmaceutical Sciences, Ritsumeikan University, Shiga, Japan

**Keywords:** development, *Drosophila*, heparan sulfate, morphogenesis, *N*-deacetylase/*N*-sulfotransferase (NDST)

## Abstract

Heparan sulfate proteoglycans (HSPGs) serve as co-receptors for growth factor signaling during development. It is well known that the level and patterns of sulfate groups of heparan sulfate (HS) chains, or HS fine structures, have a major impact on HSPG function. On the other hand, the physiological significance of other structural features of HS, including NS/NA domain organization, remains to be elucidated. A blueprint of the HS domain structures is mainly controlled by HS *N*-deacetylase/*N*-sulfotransferases (NDSTs). To analyze in vivo activities of differentially modified HS, we established two knock-in (KI) *Drosophila* strains with the insertion of mouse *Ndst1* (*mNdst1*) or *Ndst2* (*mNdst2*) in the locus of *sulfateless* (*sfl*), the only *Drosophila* NDST. In these KI lines, mNDSTs are expressed from the *sfl* locus, in the level and patterns identical to the endogenous *sfl* gene. Thus, phenotypes of *Ndst1* KI and *Ndst2*KI animals reflect the ability of HS structures made by these enzymes to rescue *sfl* mutation. Remarkably, we found that *mNdst1* completely rescued the loss of *sfl. mNdst2* showed a limited rescue ability, despite a higher level of HS sulfation compared to HS in *mNdst1* KI. Our study suggests that independent of sulfation levels, additional HS structural features controlled by NDSTs play key roles during tissue patterning.

## INTRODUCTION

Heparan sulfate proteoglycans (HSPGs) play critical roles during development and pathogenesis. Importantly, they function as co-receptors for growth factor signaling, regulating distribution and reception of secreted signaling proteins.^1–5^ In *Drosophila,* HSPGs regulate gradient formation and signaling of four key morphogen molecules, Decapentaplegic (Dpp; a *Drosophila* BMP), Wingless (Wg; a *Drosophila* Wnt), Hedgehog (Hh), and Unpaired (Upd; a ligand of the Jak/Stat pathway), as well as other secreted factors, such as FGFs, Vein (a EGF receptor ligand), and Slit.^6^

The biological function of HSPGs is dependent on both core protein and sugar moieties. During HS biosynthesis, HS undergoes sequential modification events. The first step, N-deacetylation and N-sulfation of GlcNAc units, is catalyzed by HS *N*-deacetylase/*N*-sulfotransferase (NDST). Human and mouse have four NDSTs while *Drosophila* has a single NDST gene, *sulfateless* (*sfl*).^7^ Since this reaction is essential for subsequent HS modifications, loss of *sfl* eliminates most, if not all, HS activity.^7,8^ In the following steps, C5-epimerase converts glucuronic acid residues to iduronic acid and *O*-sulfotransferases add sulfate groups to the growing chain. After the HS modification steps in the Golgi, HS can be further modified extracellularly by a family of extracellular HS 6-*O*-endosulfatases (Sulfs).^9^ Sulfs remove a subset of 6-*O* sulfate groups within the highly sulfated domains of HS.^10^ *Drosophila Sulf1* negatively regulates Wg, Hh, and EGFR signaling by reducing the number of ligand binding sites on HS.^11–14^ All these pathways are inhibited by *Sulf1* overexpression and upregulated in *Sulf1* mutants. Therefore, a higher level of HS sulfation is believed to generally upregulate growth factor signaling in *Drosophila*. Since only a fraction of potential target units are modified in each biosynthetic step, the resulting HS chains have remarkable levels of structural heterogeneity. The HS fine structures thus generated have a major impact on HSPG function.^15^

In addition to the fine structures, HS has other structural organizations. For example, chain length, net charge, and the degree/distribution of *C5*-epimerization, each of which varies depending on tissues and species. Furthermore, HS has regions that are highly sulfated (NS domains) interspersed with nonsulfated regions (NA domains).^16,17^ However, the biological importance of these structural features is poorly understood.

The formation of the NS/NA-domain structures is mainly controlled by NDSTs. When an NDST adds sulfate groups to a stretch of the growing chain, other enzymes modify that region, leading to the formation of an NS domain. When an NDST does not add *N*-sulfate groups, it will generate an NA domain. In most human and mouse tissues, Ndst1 plays a major role in the production of HS, composed of discreet NS and NA domains.^18,19^ Ndst1 knockout (KO) mice show a variety of severe defects in the development of many tissues, including lungs, eyes, skeleton, and vasculatures, resulting in high levels of lethality.^20–22^ In tissue culture cells, another NDST enzyme, named Ndst2, shows a higher activity of N-sulfation than Ndst1, producing a higher degree of N-sulfation and longer stretches of NS domains.^23,24^ This is consistent with its in vivo function. Ndst2 is responsible for the production of heparin, which lacks the alternating patterns of these domains and is characterized as a continuous NS domain.^17,25,26^ The only defect of Ndst2 KO mice is the lack of heparin in mast cells, leading to abnormal mast cell function.^25^ Thus, in vitro and in vivo studies of Ndst1 and Ndst2 revealed differential enzymatic activities and distinct developmental roles of these enzymes. However, as these NDSTs regulate structures of two different classes of polysaccharides, it is not feasible to directly compare their in vivo activities.

Here, we devised a heterologous system to measure the in vivo activities of the different HS structures generated by *Ndst1* and *Ndst2*. Mouse Ndst1 (*mNdst1*) and mouse Ndst1 (*mNdst2*) knock-in (KI) strains in the *sfl* locus allow us to compare the ability of the two mouse NDSTs to rescue the loss of *sfl*. We found that *mNdst1* shows a remarkably higher level of in vivo activity in *Drosophila* compared to *mNdst2* in spite of a lower sulfation level of HS. Our observations suggest that HS sulfation pattern design controlled by NDSTs, not simply sulfation levels, plays important roles in vivo.

## MATERIALS AND METHODS

### *Drosophila* strains

The following fly strains were used in this study: Oregon-R and *UAS*-*sfl* *RNAi* (HMS00543, BDSC #34601). Flies were raised on a standard cornmeal fly medium at 25°C, unless otherwise indicated.

### Generation of *sfl^KI:mNdst1^* and *sfl^KI:mNdst2^*

cDNA clones for *mNdst1* and *mNdst2* were obtained from Lena Kjellén (Uppsala University). To generate flies expressing *mNdst1* or *mNdst2* instead of *sfl*, we inserted the sequence containing T2A followed by *mNdst1* or *mNdst2* CDS and *sfl* 3′-UTR at the *sfl* locus using CRISPR/Cas9-mediated homology-directed repair.^27,28^ The single-guide RNA (sgRNA) was constructed by annealing 5′-CTTCGCTGTTGGACAAATACTGCC-3′ and 5′-AAACGGCAGTATTTGTCCAACAGC-3′ and ligating in the *Bbs*I-digested pU6-*Bbs*I-chiRNA plasmid (a gift from Melissa Harrison, University of Wisconsin; Kate O’Connor-Giles, Brown University; and Jill Wildonger, University of Wisconsin; Addgene #45946).^27,28^ To generate the repair template, *sfl* homology arms, T2A, *mNdst1* (or *mNdst2*) CDS, and *sfl* 3′-UTR were assembled using NEBuilder HiFi DNA Assembly Master Mix (E2611S; New England Biolabs). A mixture of 50 ng/μL of the sgRNA plasmid and 250 ng/μL of the repair template was injected into the *vasa-Cas9* embryos (BDSC #51323) by GenetiVision. The homologous recombinants were screened by PCR and verified by Sanger sequencing. The obtained strains were backcrossed to Oregon-R for five generations.

### Preparation of adult wings and legs

The right wings from female flies were dehydrated in ethanol and subsequently with xylene.^29,30^ Adult legs were boiled in 2.5 N sodium hydroxide, washed in distilled water, and dehydrated in 2-propanol.^29^ The specimens were mounted in Canada balsam (Benz Microscope, BB0020).

### RT-PCR

Expression of *sfl, mNdst1,* and *mNdst2* was analyzed by RT-PCR. *Actin5C* was used as a control. Wild-type, *Nsdt1,* and *Ndst2* adult flies were homogenized in 300 μL of TRIzol^®^ reagent (Invitrogen; 15596-026), and total RNA was isolated using Direct-zol^™^ RNA MiniPrep (Zymo Research; R2050). cDNA was synthesized from 50 ng of total RNA using SuperScript^®^ III First-Strand Synthesis System for RT-PCR (Invitrogen; 18080-051). A 0.5 μL aliquot of the cDNA synthesis reaction mixture was used to amplify the target cDNAs using the following PCR primers:

*sfl* (forward): 5′-CAAACGAAGTCATGCCCTGC-3′.

*sfl* (reverse): 5′-CAGATCCTTCAGTGCCCTCG-3′.

*mNdst1* (forward): 5′-TGGATTCCCGAGCCTTCCTA-3′.

*mNdst1* (reverse): 5′-ACCTGGTGTTCTGGAGGTCT-3′.

*mNdst2* (forward): 5′-TCCCTGTTCCTTCCAATGCC-3′.

*mNdst2* (reverse): 5′-TACCAGGAGTAGGCCCTGTC-3′.

*Act5C* (forward): 5′-GGCGCAGAGCAAGCGTGGTA-3′.

*Act5C* (reverse): 5′-GGGTGCCACACGCAGCTCAT-3′.

PCR products with expected size were analyzed by agarose gel electrophoresis.

For RT-qPCR, RNA samples were prepared from the whole third-instar larvae. cDNA was synthesized using SuperScript III First-Strand (Invitrogen). qPCR assays were performed for three independent biological replicates in a LightCycler 480 Instrument II (Roche) using LightCycler 480 SYBR Green I Master (Roche). Expression of *Ribosomal protein L23* (*RpL23rpL23*) was used for normalization. Fold changes were calculated using the ΔΔCt method.

### In situ RNA hybridization

In situ RNA hybridization was performed as described previously.^31^ Wing imaginal discs were dissected from third-instar larvae and fixed with 4% paraformaldehyde. Digoxigenin-labeled RNA probes were synthesized using a DIG RNA Labeling kit (Roche Applied Science). The hybridized probes were detected by anti-digoxigenin antibody conjugated with alkaline phosphatase (Roche Applied Science). The signal was developed by a standard protocol using 3,3-diaminobenzidine as a substrate.

### Disaccharide analysis

Approximately 10 mg of lyophilized larvae were used for analysis of HS. Briefly, crude glycosaminoglycans were obtained by extraction with 0.5% sodium dodecyl sulfate, 0.1 M NaOH, and 0.8% NaBH_4_, followed by ethanol precipitation. Chondroitin sulfate was removed from the crude glycosaminoglycan solution by chondroitinase treatment, followed by separation with a centrifugal ultrafiltration membrane, NANOSEP 3K OMEGA (Pall Life Science). The HS sample was digested with a heparitinase mixture (Seikagaku), and the resulting disaccharide species were separated using reversed-phase ion-pair chromatography. The effluent was monitored fluorometrically for postcolumn detection of HS disaccharides.^8^

## RESULTS

### *mNdst* KI alleles in the *sfl* locus

*Drosophila* produces HS, but not heparin, using a single NDST homolog, Sfl. In *sfl* mutants, all HS-dependent growth factor pathways are disrupted, and zygotic null mutants are fully lethal by late larval stages. Based on sequence homology analyses, the similarities of the Sfl amino acid sequence to those of mNdst1 and mNdst2 are comparative (Supporting Information S1: Figure S1). For example, sequence alignment using EMBOSS Water with the Smith–Waterman algorithm revealed 70.2% sequence similarity between Sfl-Ndst1 and 71.2% between Sfl-Ndst2. Thus, structurally, mNdst1 and mNdst2 are equally similar to (and distant from) Sfl.

**FIGURE 1:**
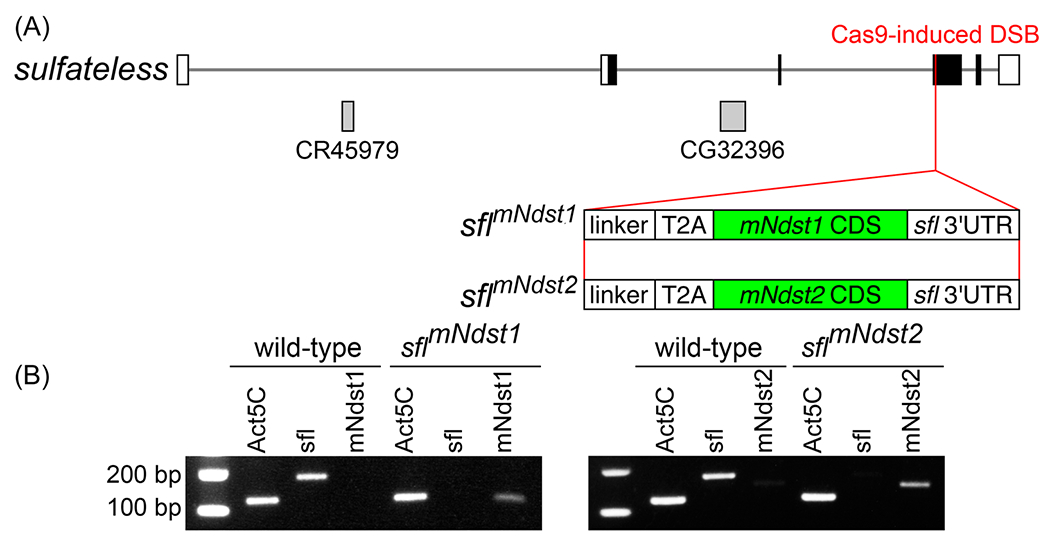
Organization of the *sfl* locus and generation of *mNdst1* and *mNdst2* knock-in (KI) alleles. (A) Schematic of *mNdst1* and *mNDST2* inserted into the *sfl* locus. Using CRISPR/Cas9 gene editing, mouse Ndst1 (*mNdst1*) and Ndst2 (*mNdst2*) were inserted into the *sfl* locus of the *Drosophila* genome. Each insertion also included a linker sequence, *Thosea asigna* virus 2A DNA, which functioned to help the messenger RNA (mRNA) translation, and *sfl* 3′-untranslated region. (B) Reverse transcription-polymerase chain reaction showing expression of sfl, mNdst1, and mNdst2 in *Ndst1* (left) and *Ndst2* (right) KI alleles. *Ndst1* and *Ndst2* flies express respective *Ndst* mRNAs but not *sfl. Act5C* was used as an internal control and is expressed in both the wild-type and two KI strains.

We devised a system to compare the in vivo activities of HS modified by different NDSTs using the *Drosophila* model. We generated two KI *Drosophila* strains with the insertion of *mNdst1* or *mNdst2* in the endogenous *sfl* locus via CRISPR/Cas9 gene editing (Figure 1A). To achieve expression of *mNdst1* and *mNdst2* in the patterns of the endogenous *sfl* gene, we employed the *Thosea asigna* virus 2A (T2A) in-frame fusion technology, which utilizes a “ribosomal skipping” mechanism of the T2A peptide.^32,33^ We inserted T2A–mNdst complementary DNA (cDNA) constructs after Tyr_315_ of the *sfl* protein coding sequence in frame, and thus mNdst cDNAs are transcribed as a fusion RNA. T2A is a viral ribosomal skipping site that terminates translation at the beginning of the peptide and reinitiates it after the site, producing a truncated Sfl peptide and full-length mNdst enzymes. mNdst protein coding sequences are followed by the 3′-untranslated region (3′-UTR) of *sfl* messenger RNA (mRNA), including the polyadenylation sequence. Therefore, the *sfl* genomic sequence after the insertion site is not transcribed. As a result, no functional Sfl protein is produced in these transgenic strains (Supporting Information S1: Figure S1). Instead, the *mNdsts* are expressed from the *sfl* locus in the level and patterns identical to the endogenous *sfl* gene in wild type. Reverse transcription-polymerase chain reaction (RT-PCR) analyses verified the loss of *sfl* mRNAs and expression of respective *Ndst* genes in these KI alleles (Figure 1B). In situ RNA hybridization showed that *sfl* mRNA is uniformly expressed in the wild-type larval imaginal discs and central nervous system (CNS), consistent with the fact that *sfl* is an essential gene required for the biosynthesis of ubiquitous HS. We detected transcripts of *Ndst1* in *Ndst1* KI and *Ndst2* in *Ndst2* KI at indistinguishable levels and patterns from *sfl* (Supporting Information S1: Figure S2A–H). RT-quantitativePCR (RT-qPCR) also confirmed comparative levels of expression of *sfl* in wild type, *Ndst1* in *Ndst1* KI, and *Ndst2* in *Ndst2* KI (Supporting Information S1: Figure S2I). These novel *Drosophila* strains, *sfl^KI:mNdst1^* and *sfl^KI:mNdst2^*, are referred to as *Ndst1* and *Ndst2* strains, respectively, in this paper. This heterologous system allows the first systematic approach to compare in vivo activities of distinct HS structures modified by different NDSTs.

### Disaccharide analyses of HS from *Ndst* KI alleles

As detailed below, we found that *mNdst1* and *mNdst2* rescue the loss of *sfl* in these KI alleles, and animals survive to adult stages. To determine if Ndst1 and 2 differentially modify HS in *Drosophila*, as has been observed in vitro,^24^ we analyzed the structures of “*Drosophila* HS” modified by mNdst1 and 2 by disaccharide analysis. Briefly, HS was purified from third-instar larvae and completely digested into disaccharides by heparitinase. The resultant disaccharide species were separated and quantified by reversed-phase ion-pair chromatography with a postcolumn detection system.^8,11,34,35^

**FIGURE 2:**
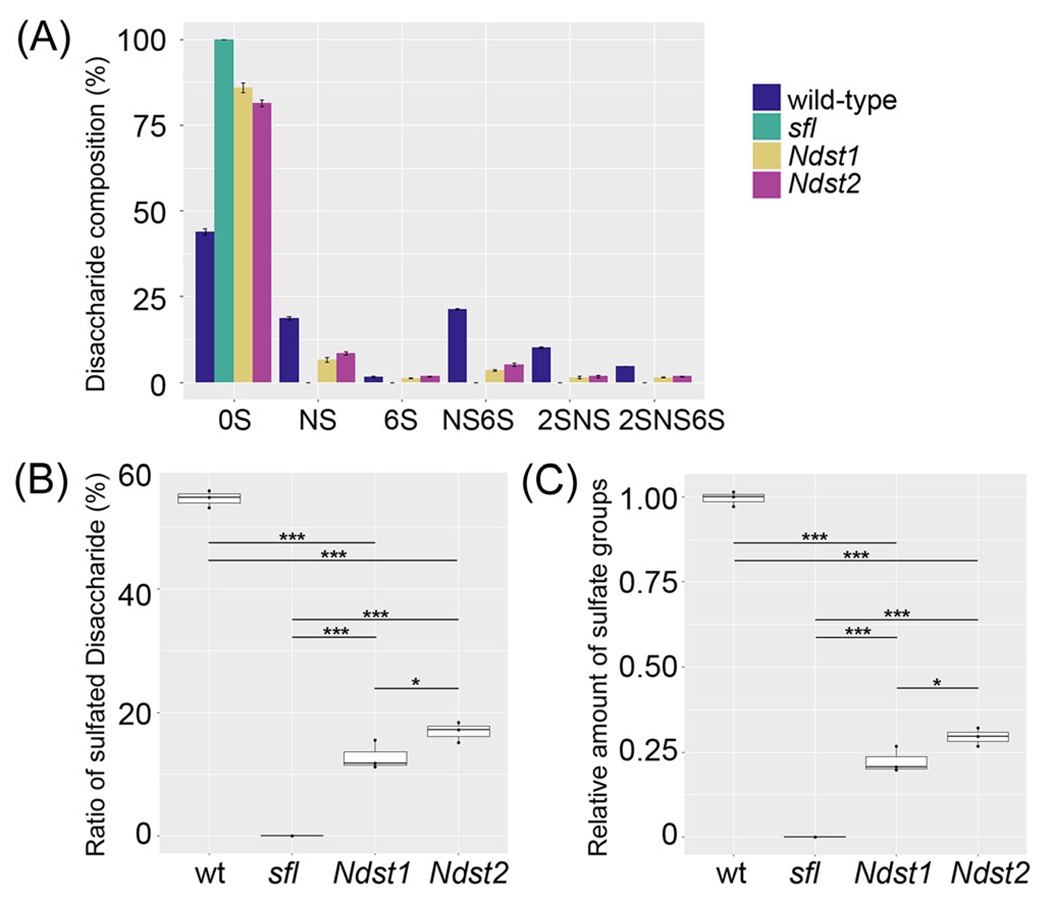
Heparan sulfate (HS) disaccharide profiling of mouse *Ndst1* (*mNdst1*) and *mNdst2* knock-in alleles. (A) Graphical depiction of disaccharide composition of HS from wild-type (wt) (dark blue), *sfl* (green), *Ndst1* (yellow) and *Ndst2* (magenta), represented as percent of total HS. Sulfated disaccharide units were absent in HS from *sfl* mutants. (B) The percentage of sulfated disaccharides in wt, *sfl, Ndst1*, and *Ndst2*. The sulfation levels of HS in *Ndst1* and *Ndst2* are significantly lower than wt. *Ndst2* HS has significantly higher levels of sulfated disaccharides compared to that from *Ndst1*. (C) Relative amount of total sulfate groups in each genotype. The relative amounts were calculated by multiplying the ratios of monosulfated (NS, 6S), disulfated (NS6S, 2SNS), and trisulfated (2SNS6S) disaccharides by 1, 2, and 3, respectively. NS, ΔUA-GlcNS; NS2S, ΔUA2S-GlcNS; NS6S, ΔUA-GlcNS6S; 2SNS6S, ΔUA2S-GlcNS6S; 0S, ΔUA-GlcNAc; 6S, ΔUAGlcNAc6S. *p < 0.05; ***p < 0.001 (two-sided, unpaired *t*-test).

As was previously shown,^36^ HS from *sfl* mutant larvae lacks sulfated disaccharide units (Table 1 and Figure 2A). mNdst1 and 2 partially rescued this phenotype, producing sulfated disaccharide species, including the tri-sulfated unit (ΔUA2S-GlcNS6S or 2SNS6S). However, the sulfation levels of HS in these KI alleles were significantly lower than in wild type. The proportion of sulfated disaccharides of *Ndst1* and *Ndst2* were 23% and 31% of wild type, respectively (Table 1 and Figure 2B).

Importantly, consistent with previous in vitro observations,^23,24^ *Ndst2* HS has significantly higher levels of sulfated disaccharides compared to that from *Ndst1* (Table 1 and Figure 2B). The average percentages are higher in *Ndst2* than *Ndst1* for all sulfated disaccharides. The percentage of total sulfated disaccharides was significantly higher in *Ndst2* than *Ndst1* (Table 1). We also calculated the relative amounts of total sulfate groups in each genotype by multiplying the ratios of monosulfated (NS, 6S), disulfated (NS6S, 2SNS), and trisulfated (2SNS6S) disaccharides by 1, 2, and 3, respectively (Figure 2C). This value was also significantly higher in *Ndst2*, indicating that *Ndst2* HS contains more sulfate groups compared to *Ndst1*. Our in vivo results confirmed that these two enzymes differentially modify HS in *Drosophila*. More specifically, mNdst2 shows a higher activity to add sulfate groups on HS compared to mNdst1 in this heterologous system.

Comparison of total HS (ng/mg dry tissue) recovered in disaccharide analyses showed no significant difference in the amount of HS from wild-type and the two *Ndst* KI alleles (Table 1 and Figure S3). Interestingly, the amount of unsulfated polysaccharide (heparosan) from *sfl* mutants was lower than HS from other genotypes. As an NDST affects HS chain length,^37^ this may reflect a shorter chain length of *sfl* heparosan. Future studies are needed to reveal the molecular basis for the reduced recovery of heparosan.

### *mNdst1* fully rescues the lethality of *sfl*

**FIGURE 3:**
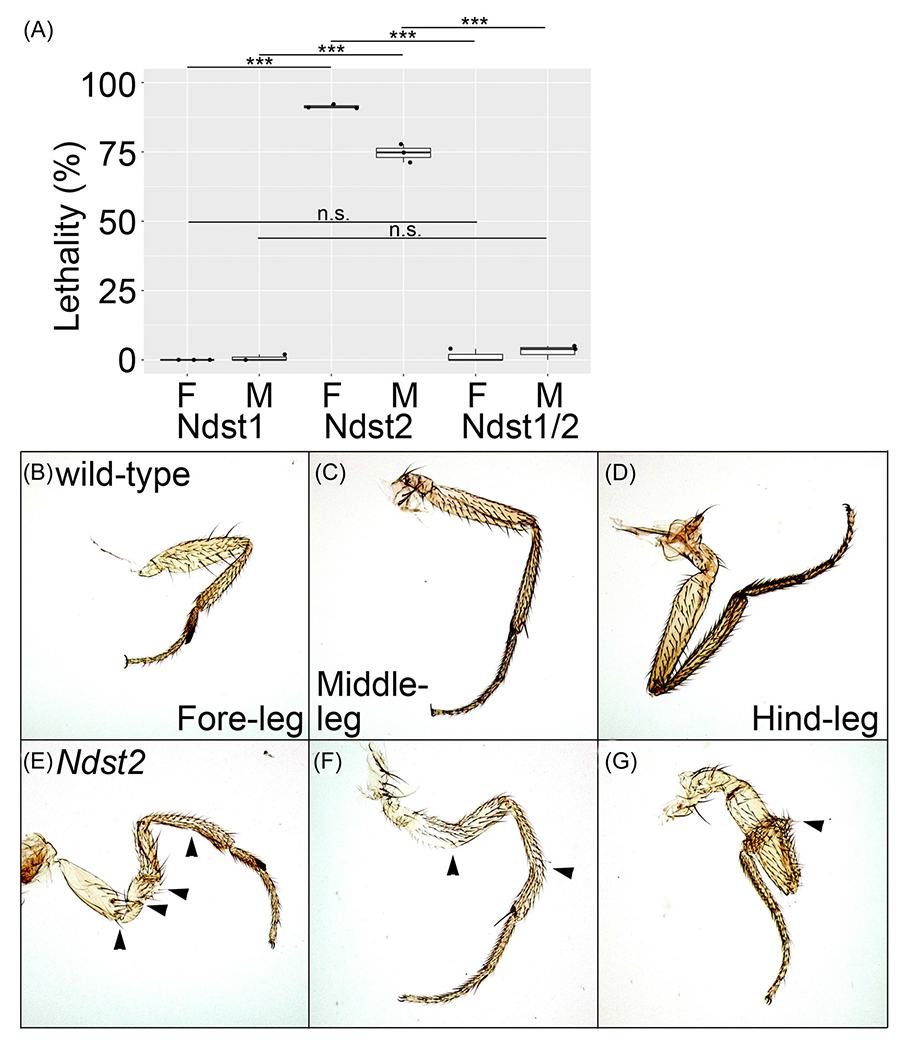
Mouse *Ndst1* (*mNdst1*) rescues the lethality of *sfl* mutant. (A) Lethality of *mNdst1* and *mNdst2* knock-in alleles. The lethality rate of female (F) or male (M) of *Ndst1* homozygotes (Ndst1), *Ndst2* homozygotes (Ndst2), and *Ndst1/Ndst2* heterozygotes (Ndst1/2) were calculated by three sets of independent experiments. More than 230 flies per set for each sex were counted for all genotypes. Boxes indicate the 25th–75th percentiles, and the median is marked with a line. The whiskers extend to the highest and lowest values within 1.5 times the interquartile range. (B–G) Forelegs (B and E), middlelegs (C and F), and hindlegs (D and G) are shown for wild type (B–D) and *Ndst2* KI (E–G). Arrowheads mark leg patterning defects in *Ndst2* legs, such as bending and twisting. n.s., not significant; ***p < 0.001 (two-sided, unpaired *t*-test).

To examine in vivo activities of differentially modified HS by *Ndst1* and *Ndst2*, we asked which gene shows a higher ability to rescue the lethality of *sfl* mutant. Our lethality assay showed that both genes can rescue it but to significantly different degrees. We found that the lethality of *sfl* mutants is completely restored by *Ndst1* (Figure 3A). This is remarkable given that the HS sulfation level of these animals is only 23% of wild type. Thus, the minimal level of HS sulfation required for normal viability is unexpectedly low.

In contrast, *Ndst2* KI shows a high level of lethality (91.4% for females, 74.6% for males) (Figure 3A), indicating a limited ability of the rescue. We found that the lethality was also fully rescued in *Ndst1/Ndst2* heterozygotes (Figure 3A), which suggests a dominant effect of Ndst1’s ability to complement *sfl* function.

### *Ndst2* KI shows leg patterning defects

*Ndst2* homozygotes die at various stages, including the lethality at late pupal/pharate adult stages due to eclosion failure. We found that dead pharate adults dissected from pupal cases have malformed legs. In addition, *Ndst2* adult flies that successfully eclosed also show morphological defects in their legs. *Ndst2* adults exhibit patterning abnormalities in fore, middle, or hindlegs, such as twisting and bending of the appendages (Figure 3B–G). In contrast, these defects are not observed in *Ndst1*. The leg patterning phenotypes explain the eclosion failure and appear to contribute to the lethality of *Ndst2* homozygotes to some extent. Except for the legs, *Ndst2* survivors do not show any obvious major defect in other adult organs, suggesting that leg morphogenesis is particularly sensitive to a change in N-sulfation patterns of HS. At this point, however, the molecular basis for this sensitivity of leg morphogenesis to *Ndst2* KI is unknown.

### Wing morphology of *Ndst1* KI and *Ndst2* KI adult survivors

**FIGURE 4:**
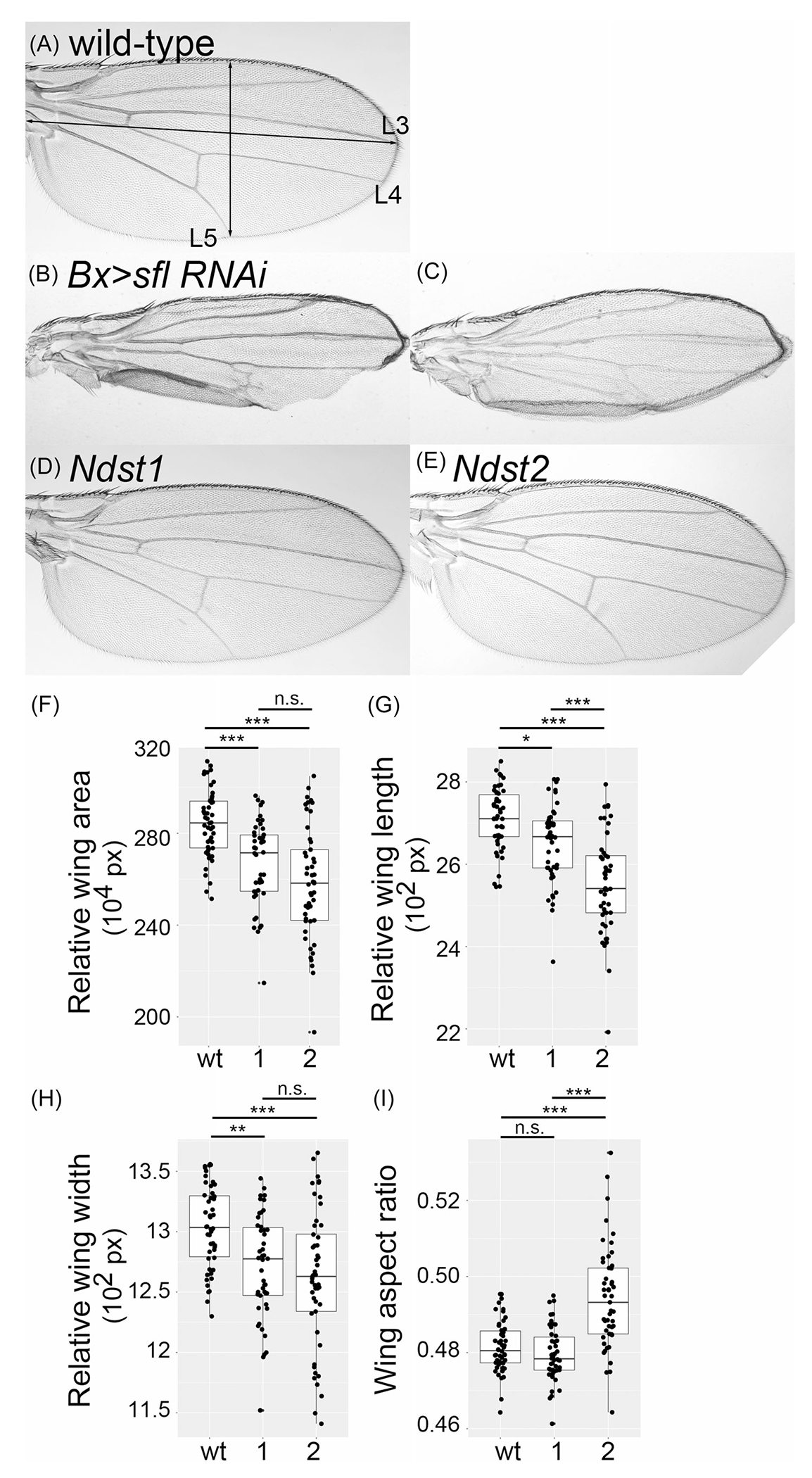
Wing morphology of *Ndst1* knock-in (KI) and *Ndst2* KI adult survivors. (A–E) Adult wings of wild type (wt) (A), *Bx>sfl RNAi* (RNA interference) (B and C), *Ndst1* (D), and *Ndst2* (E) are shown. Arrows in (A) show the length and width of the wing. (F–I) Quantification of relative wing area (F), wing length (G), wing width (H), and wing aspect ratio (width/length, I) for wt, *Ndst1* (1), and *Ndst2* (2) wings. At least 50 wings were measured for each genotype. Values are shown by pixels for the relative length and width, and by pixel^2^ for the relative area. n.s., not significant; *p < 0.05; **p < 0.01; ***p < 0.001 (two-sided, unpaired *t*-test).

To further examine the effect of HS sulfation design mediated by Ndsts, we compared in vivo patterning activities of *Ndst1* and *Ndst2* during wing development, a model system extensively used for morphogen function studies. When *sfl* function is compromised by RNA interference (RNAi) knockdown specifically in the developing wing using *Bx-Gal4*, a commonly used wing Gal4 driver, knockdown animals (*Bx>sfl RNAi*) show a variety of wing phenotypes, including loss of anterior and posterior crossveins, wing cell growth defects, wing notching, ectopic venation, and blistered wing (Figure 4B,C). These patterning defects are consistent with the idea that *sfl* is required for Hh, Dpp, and Wg morphogen signaling during wing development.^7,38–40^ We found that overall wing patterning of adult survivors of *Ndst1* and *Ndst2* is quite normal (Figure 4D,E), indicating a remarkable ability of both genes to rescue the loss of *sfl* in the wing. It is particularly interesting that *Ndst2* wings do not show any obvious morphological defects (Figure 4E) despite its high level of lethality.

However, quantitative analyses of wing morphology parameters of *Ndst1* and *Ndst2* adults revealed that in general, *Ndst1* shows a stronger activity to rescue *sfl* patterning defects compared to *Ndst2* (Figure 4F–I). The following parameters were used for comparison: wing area, length, width, and aspect ratio (width/length). Wing length was measured as the distance between the distal edge of the third longitudinal vein (L3) to the wing hinge (horizontal arrow in Figure 4A). Wing width was measured as the shortest distance between the posterior edge of the fifth longitudinal vein (L5) to the wing margin (vertical arrow in Figure 4A).

We found that both *Ndst1* and *Ndst2* wings are smaller, with reduced length and width (Figure 4F). *Ndst2* is more severely affected than *Ndst1* in the wing area, length, and width (Figure 4F–H). Interestingly, however, *Ndst2* wings are particularly short in length compared to both wild type and *Ndst1* (Figure 4G). As a result, the wing aspect ratio (width/length) of *Ndst2* wings is significantly higher than wild type and *Ndst1*, which means that *Ndst2* wings are rounder (Figure 4I). On the other hand, *Ndst1* wings have a wild-type wing aspect ratio, indicating that length and width are proportionally reduced.

Taken together, *Ndst* KI alleles show not only different levels of rescue of *sfl* mutation but also distinct patterns of complementation. During wing development, some morphogen pathways regulate growth and patterning along the A–P axis (Hh and Dpp), and others affect the D–V axis (Wg). Therefore, our data suggest a possibility that HS designs differentially modified by NDSTs may uniquely affect distinct morphogen pathways.

### Wing margin phenotypes of *Ndst1* KI and *Ndst2* KI adult flies

**FIGURE 5:**
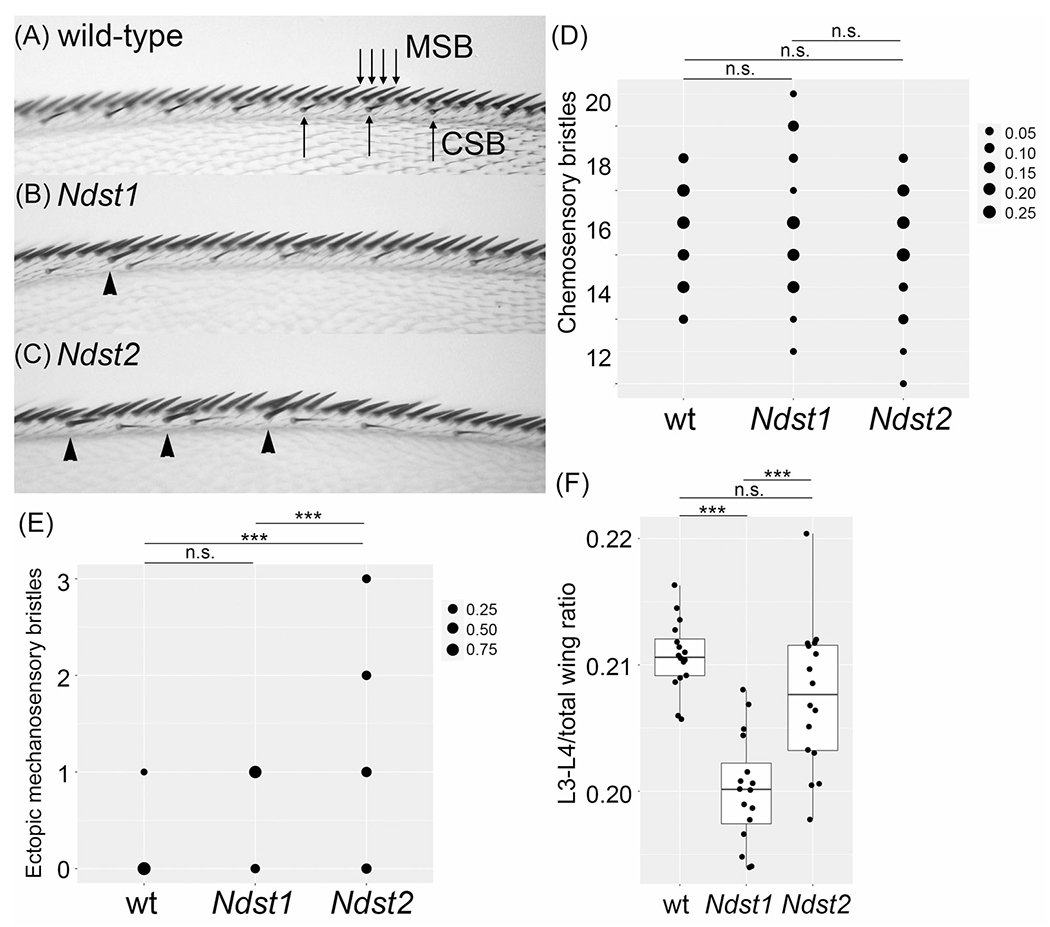
Wing margin phenotypes and the ratio of the third/fourth longitudinal vein (L3/L4) domain of *Ndst1* knock-in (KI) and *Ndst2* KI adult flies. (A–C) The anterior wing margin of wild-type (wt) (A), *Ndst1* (B), and *Ndst2* (C) are shown. Mechanosensory bristles (MSBs) and chemosensory bristles (CSBs) are marked by arrows. Ectopic MSBs are shown by arrowheads in (B) and (C). (D and E) Density plots showing quantification of CSBs (D) and ectopic MSBs (E) of wt, *Ndst1*, and *Ndst2* wings. (F) Quantification of the L3–L4 area ratio in wt, *Ndst1*, and *Ndst2* wings. n.s., not significant; ***p < 0.001 (two-sided, unpaired *t*-test).

We next determined if *mNdst1* and *mNdst2* show different activities on the wing margin formation, an event controlled by Wg signaling. The anterior wing margin bears several rows of different types of sensory organs: thick and short mechanosensory bristles at the edge and thinner chemosensory bristles slightly posterior to the edge (Figure 5A^41^). Dally, one of the two *Drosophila* glypicans, acts as a Wg co-receptor, and the loss of *dally* substantially decreases the number of these bristles. On the other hand, knockdown of *dally-like protein* (*dlp*), the second glypican gene, results in increased Wg signaling near the wing margin.^42–46^ This leads to the formation of ectopic mechanosensory bristles that are shifted from their normal position, the edge of the wing.^42^

We found that wings of both *Ndst1* and *Ndst2* adult survivors have a normal number of chemosensory bristles at the anterior wing margin (Figure 5A–D). Interestingly, however, *Ndst2* KI wings bear ectopic mechanosensory bristles, similar to *dlp* knockdown (Figure 5C,E). *Ndst1* occasionally showed this phenotype, but the difference between wild-type and *Ndst1* wings was not statistically significant (Figure 5B,E). These observations suggest that HS modified by mNdst2 may induce an abnormally higher level of Wg signaling at the wing margin.

### Different effects of *Ndst1* and *Ndst2* on the ratio of the L3/L4 domain

Although *Ndst1* shows much higher activities to rescue the loss of *sfl* in most contexts, there is a phenotype in which *Ndst2* may function better than *Ndst1*. Hh signaling regulates the growth and patterning of the central region of the wing, and manipulation of this pathway affects the spacing between longitudinal wing veins L3 and L4 (Figure 4A). Thus, the ratio of the area between L3 and L4 (L3/L4 domain) in the total wing area reflects Hh signaling activity.^47^

We found that the L3/L4 domain ratio is significantly reduced in *Ndst1* wings, suggesting that *Ndst1* shows a low activity to rescue reduced Hh signaling of the *sfl* mutants (Figure 5F). In contrast, in *Ndst2* wings, the L3/L4 ratio was not significantly different from wild type. Thus, unlike other parameters, *Ndst2* appears to show a higher ability to rescue an Hh-dependent process compared to *Ndst1*.

## DISCUSSION

The first step of HS modification is catalyzed by NDSTs, which generate a blueprint of domain structures of HS. In mammals, HS has alternating patterns of a highly sulfated NS domain and an unsulfated NA domain. Different NDSTs generate distinct NS/NA domain structures. For example, Ndst1 (which is critical for producing HS) makes clearly distinct NS/NA domains, while Ndst2 (which produces heparin) continuously adds sulfate groups to make a chain composed of a single NS domain, without NA domains.

It is worth noting that Ndst2 can synthesize HS-like structures in vivo.^48,49^ In wild-type mouse embryos, Ndst2 is present in the liver but it does not contribute to HS structure. However, in Ndst1 KO liver cells, where Ndst2 is the only NDST isoform expressed, it forms the same HS structures as wild type. The observation suggested that the GAGosome, an active complex of the HS biosynthetic/modifying enzymes, preferentially incorporates Ndst1, but Ndst2 is recruited to the GAGosome in the absence of Ndst1.

In vivo functions of *Ndst1* or *Ndst2* have been extensively studied using KO mouse strains.^18,20–22,25^ Ndst1 KO mice are perinatal lethal and show developmental defects in the respiratory, nervous, skeletal, and vascular systems.^22^ The broad array of phenotypes from Ndst1 KO mice include—but are not limited to—abnormal lungs resembling respiratory distress syndrome in humans, cerebral hypoplasia and patterning defects, axon guidance defects, neural tube closure failure, the loss of neural crest cell-derived elements, the loss of olfactory bulbs, delayed ossification, defects of the mandible, and delayed pericyte recruitment.^18,21,50,51^ Conversely, Ndst2 KO mice are viable and only show one clear phenotype: disrupted mast cells due to the loss of sulfated heparin synthesized by NDST2.^18,52^ The lack of lethality and phenotypes observed in Ndst2 KO mice is striking given that Ndst2, along with Ndst1, is expressed throughout the body in mice.^18^ The difference between Ndst1 KO and Ndst2 KO mice provides clear evidence that Ndst1 and Ndst2 function distinctly in heparan sulfate biosynthesis.

However, since Ndst1 or Ndst2 contribute to completely different biological roles, the mouse KOs cannot allow us to directly compare the activities of HS differentially modified by these enzymes. To fill this knowledge gap, we generated two KI strains with an insertion of *mNdst1* or *mNdst2* in the endogenous *sfl* locus using the T2A in-frame fusion technology. This methodology has been successfully used to generate KI transgenic strains in *Drosophila*, including with high-throughput genome-wide platforms.^53,54^ Our heterologous system provides a unique opportunity to study the in vivo activities of HS differentially modified by mNDST1 and mNDST2. Interestingly, the ability of these mouse genes to rescue *sfl* mutants was significantly different. *Ndst1* almost perfectly rescues the lethality and major morphological defects of *sfl* mutants. In contrast, *Ndst2* shows limited ability to rescue them. Our findings were remarkable for two reasons. First, HS in *Ndst1* bears only 23% of the wild-type sulfation level (Table 1 and Figure 2), but exhibits virtually wild-type in vivo activities during tissue patterning. Second, although *Ndst2* KI HS has higher compositions of sulfated disaccharide species compared to *Ndst1*, consistent with their enzymatic activities, it shows a significantly lower activity to rescue *sfl*. Our observations suggest a possibility that HS structures controlled by NDSTs, rather than simply sulfation levels, are important for in vivo patterning activities of HS.

Although *Ndst1* generally showed a significantly higher ability to rescue *sfl* compared to *Ndst2*, there were a few exceptions. First, the wing aspect ratio (width/length) of *Ndst1* and *Ndst2* are different, making *Ndst2* wings rounder. Second, the L3/L4 domain ratio of *Ndst1* wings is more significantly reduced. These observations suggest that HS structures modified by Ndst1 and 2 may have unique and differential effects on distinct morphogen pathways.

We still do not know whether the NS/NA domain structure is the source of differential in vivo activities between *Ndst1* and *Ndst2*. What specific HS structural features are differentially modified by these enzymes in *Drosophila* remains to be determined. It had been difficult to analyze *Drosophila* HS structures, with the exception of disaccharide composition, mainly due to the difficulty of metabolic radiolabeling of HS in vivo using *Drosophila* animals.^11–13,31,34,55,56^ We recently established a novel system to overcome this obstacle.^35^ This platform is composed of two steps: generation of immortalized cell lines derived from existing *Drosophila* strains^57–59^ and metabolic radiolabeling and structural analysis of HS from these established cell lines.^35^ This system allows us to connect in vivo phenotypic information of *Drosophila* strains and detailed HS structural features. Future studies using this strategy will be able to identify the differences in HS structures between *Ndst1* and *Ndst2* animals. Thus, our study opens up a new strategy of “in vivo structure–function analysis” of HS.

## Supplementary Material

Supplemental Material

## Figures and Tables

**TABLE 1:** Disaccharide analyses of HS from wild-type, *sfl, Ndst1*, and *Ndst2* animals.

Genotype	HS (unsaturated disaccharide, %)						Total HS (ng/mg dry tissue)
	NAc	NS	NAc6S	NS6S	2SNS	2SNS6S	
Wild type	43.8 ± 1.7	18.6 ± 0.9	1.6 ± 0.4	21.2 ± 0.4	10.2 ± 0.4	4.6 ± 0.1	18.1 ± 1.2
*sfl*	100.0	ND	ND	ND	ND	ND	13.6 ± 0.7
*Ndst1*	86.0 ± 2.5	6.5 ± 1.3	1.2 ± 0.2	3.5 ± 0.4	1.4 ± 0.5	1.4 ± 0.2	17.7 ± 1.3
*Ndst2*	81.4 ± 1.8	8.5 ± 0.8	1.7 ± 0.1	5.1 ± 0.7	1.7 ± 0.4	1.7 ± 0.1	15.2 ± 1.6

*Note*: Disaccharide composition of HS is shown for each respective genotype. The values are given as mol% of total disaccharides, and represent mean ± SD from three independent experiments. The rightmost column shows the amount of total HS (ng/mg dry tissue) in each genotype. Graphical depictions of the disaccharide composition and total HS are shown in Figure 2 and Supporting Information S1: Figure S3, respectively.

Abbreviation: HS, heparan sulfate; NAc, ΔUA-GlcNAc; NAc6S, ΔUAGlcNAc6S; ND, not detectable; NS, ΔUA-GlcNS; NS6S, ΔUA-GlcNS6S; 2SNS, ΔUA2S-GlcNS; 2SNS6S, ΔUA2S-GlcNS6S.

## Abbreviations:

cDNA: complementary DNA
CNS: central nervous system
dlp: Dally-like protein
Dpp: Decapentaplegic
EGF: epidermal growth factor
FGF: fibroblast growth factor
GlcNAc: N-Acetylglucosamine
Hh: Hedgehog
HS: heparan sulfate
HSPGs: heparan sulfate proteoglycans
KI: knock-in
KO: knockout
L3: longitudinal wing vein 3
L4: longitudinal wing vein 4
L5: longitudinal wing vein 5
mNDST1: mouse N-deacetylase/N-sulfotransferase 1
mNDST2: mouse N-deacetylase/N-sulfotransferase 2
mRNA: messenger RNA
NA: N-acetylated
NDSTs: N-deacetylase/N-sulfotransferases
NS: N-sulfated
sfl: Sulfateless
Sulfs: 6-O-sulfatases
RNAi: RNA interference
RT-PCR: reverse transcription-polymerase chain reaction
RT-qPCR: reverse transcription quantitative polymerase chain reaction
sgRNA: single-guide RNA
T2A: Thosea asigna virus 2A
Upd: Unpaired
UTR: untranslated region
Wg: Wingless
