# Supplementary material for: In vivo activities of heparan sulfate differentially modified by NDSTs during development
Source: Proteoglycan Res. Author manuscript; Available in PMC 2024 Apr 12. (PMC11011245; doi:10.1002/pgr2.17)
Supplement: Supplemental Material [file NIHMS1971439-supplement-Supplemental_Material.pdf]

```
mNDST1          ------------------------------------------------------------
mNDST2          ------------------------------------------------------------
Sfl             MTISGGNQHNNNANRKYEKLIKQPQMQFGSSVTGTQTDVDSCRDADADANAVRQDFSNFN

mNDST1          ------------------------------------------------------------
mNDST2          ------------------------------------------------------------
Sfl             KHFGNGHAITDRTMLLRLEDDVTTAAGIVTYKGKSNGNGNGNGNGSIGSISLDFNGSPTS

mNDST1          ----------------------------------MPALACLRRLCRHLSPQAVLFLLFVF
mNDST2          ----------------------------------MLQLWKVVRPARQLELHRLILLLIGF
Sfl             STSIGIASGSSSNTHLASGGGVGGIGGSEPAGWMCHCCNLIARRCFGINVRRCVLALLAI
                                                        : * .  :. :  :: *: :

mNDST1          CLFSVFVSAYYLYG---WNRGLEPSADAS--ESDCGDPPPVAPSRLLPIKPVQAVAPSRT
mNDST2          SLVSMGFLAYYVST---SPKAKEPLPLPLGDCSSSGAAGPGPARPPVPPRPQRPPETTRT
Sfl             TMVSIFYYTHYVDTGVFNGLIQRDTHPAPIINCRMINSGGKHIRNASPAPDHRSEARLRI
                 :.*:   ::*:          .    .    .    .         *    :.    *

mNDST1          DPLVLVFVESLYSQLGQEVVAILESSRFKYRTEIAPGKGDMPTLTDKGRGRFALIIYENI
mNDST2          EPVVLVFVESAYSQLGQEIVAILESSRFRYSTELAPGRGDMPTLTDHTHGRYVLVIYENL
Sfl             DPKVLVFVETTYSGLGRDIAELLVYNRIKYKIEVAG--KSLPVLTNLDKGRYGVIVFENL
                :* ******: ** **:::. :*  .*::*  *:*    .:*.**:  :**: ::::**:

mNDST1          LKYVNLDAWNRELLDKYCVAYGVGIIGFFKANENSLLSAQLKGFPLFLHSNLGLKDCSIN
mNDST2          LKYVNLDAWSRELLDRYCVEYGVGIIGFFRAREHSLLSAQLKGFPLFLHSNLGLRDYQVN
Sfl             DKYLNMDKWNRELLDKYCREYSVGIVGFVSPSEETLVGAQLRDFPLFVNTNLRLRDASLN
                 **:*:* *.*****:**  *.***:**. . *.:*:.***:.****:::** *:* .:*

mNDST1          PKSPLLYVTRPSEVEKGVLPGEDWTVFQSNHSTYEPVLLAKTRSSESIPHLGADAGLHAA
mNDST2          PSAPLLHLTRPSRLEPGPLPGDDWTIFQSNHSTYEPVLIASHRPAELSMPG---PVLRRA
Sfl             PLSSVLRLTRAGETAWGALPGDDWAVFQHNHSTYEPVEWAQRNTQEYPADS---VGQVQL
                * :.:* :**...   * ***:**::** ********  *. .. *

mNDST1          LHATVVQDLGLHDGIQRVLFGNNLNFWLHKLVFVDAVAFLTGKRLSLPLDRYILVDIDDI
mNDST2          RLPTVVQDLGLHDGIQRVLFGHGLSFWLHKLVFVDAVAYLTGKRLCLDLDRYILVDIDDI
Sfl             PLTTVLQDRGQLDGIQRVLFGSSLRFWLHRLVFLDALSYLSHGQLSLNLERMILVDIDDI
                  .**:** *  ********* .* ****:***:**:::*:  :*.* *:* ********

mNDST1          FVGKEGTRMKVEDVKALFDTQNELRTHIPNFTFNLGYSGKFFHTGTDAEDAGDDLLLSYV
mNDST2          FVGKEGTRMKVADVEALLTTQNKLRTLVPNFTFNLGFSGKFYHTGTEEEDAGDDMLLKHR
Sfl             FVGEKGTRLRPDDVRALIATQKNIAAMVPGFRFNLGFSGKYYHHGTREENLGDDFLLQNV
                ***::***::  **.**: **::: : :*.* ****:***::* **  *: ***:**.

mNDST1          KEFWWFPHMWSHMQPHLFHNQSVLAEQMALNKKFAVEHGIPTDMGYAVAPHHSGVYPVHV
mNDST2          REFWWFPHMWSHMQPHLFHNRSVLADQMRLNKQFALEHGIPTDLGYAVAPHHSGVYPIHS
Sfl             QEFNWFSHMWKHQQPHLYDNLTLLMAEMHLNYAFAVDHNIPTDSGYSISPHHSGVYPAHE
                :** **.***.* ****:.* ::*  :* **  **::*.**** **:::******** *

mNDST1          QLYEAWKQVWGIRVTSTEEYPHLKPARYRRGFIHNGIMVLPRQTCGLFTHTIFYNEYPGG
mNDST2          QLYEAWKSVWGIQVTSTEEYPHLRPARYRRGFIHNGIMVLPRQTCGLFTHTIFYNEYPGG
Sfl             LLYMAWKKVWNVKVTSTEEYPHLRPARLRRGFIHRNIMVLPRQTCGLFTHTMYIDRYPGG
                 ** ***.**.::**********:*** ******..***************:: :.****
```

```
mNDST1          SSELDKIINGGELFLTVLLNPISIFMTHLSNYGNDRLGLYTFKHLVRFLHSWTNLRLQTL
mNDST2          SRELDRSIRGGELFLTVLLNPISVFMTHLSNYGNDRLGLYTFESLVRFLQCWTRLRLQTL
Sfl             RDKLDESIQGGELFQTIVYNPINIFMTHMSNYGSDRLALYTFQSVIKFLQCWTNLKLASA
                  :**. *.***** *:: ***.:****:****.***.****: :::**:.**.*:* : 

mNDST1          PPVQLAQKYFQIFSEEKDPLWQDPCEDKRHKDIWSKEKTCDRFPKLLIIGPQKTGTTALY
mNDST2          PPVPLAQKYFELFPQERSPLWQNPCDDKRHKDIWSKEKTCDRLPKFLIVGPQKTGTTAIH
Sfl             PPVQLAEMYFRLHPEEVDPVWGNPCDDVRHKKIWSKTKNCDSLPKFLVIGPQKTGTTALY
                *** **: **.:..:* .*:* :**:* ***.**** *.** :**:*::*********::

mNDST1          LFLGMHPDLSSNYPSSETFEEIQFFNGHNYHKGIDWYMEFFPIPSN-----------TTS
mNDST2          FFLSLHPAVTSSFPSPSTFEEIQFFNGPNYHKGIDWYMDFFPVPSN-----------AST
Sfl             TFLSMHGSIASNIASPETFEEVQFFNGNNYYRGLDWYMDFFPSESLPNTSSPMPTQLGSP
                 **.:*  ::*. .*..****:***** **::*:****:***  *             :.

mNDST1          DFYFEKSANYFDSEVAPRRAAALLPKAKILSILINPADRAYSWYQHQRAHDDPVALKYTF
mNDST2          DFLFEKSATYFDSEVVPRRGAALLPRAKIITVLINPADRAYSWYQHQRAHGDPIALNYTF
Sfl             RFMFEKSATYFDGEAVPKRSHALLPHAKIVTILISPAKRAYSWYQHQRSHGDVIANNYSF
                 * *****.***.*..*:*. ****:***:::**.**.**********:*.* :* :*:*

mNDST1          HEVITAGPDASSKLRALQNRCLVPGWYATHIERWLSAFHANQILVLDGKLLRTEPAKVMD
mNDST2          YQVISASSQAPLLLRSLQNRCLVPGYYSTHLQRWLTYYPSGQLLIMDGQELRVNPAASME
Sfl             YQVITASDSAPRALKDLRNRCLNPGKYAQHLEHWLAYYPAQQLHIIDGEQLRLNPIDVMN
                ::**:*. .*.  *: *:**** ** *: *:::**: : : *: ::**: ** :*   *:

mNDST1          TVQKFLGVTSTVDYHKTLAFDPKKGFWCQLLEGGKTKCLGKSKGRKYPEMDLDSRAFLKD
mNDST2          IIQKFLGITPFLNYTRTLRFDEDKGFWCQGLEGGKTRCLGRSKGRRYPDMDMESRLFLTD
Sfl             ELQRFLKIQPLLDYSNHLRYDVKKGFYCQAVSEKRNKCLGKSKGRQYPAMDERSAKLLQR
                 :*:** : . ::* . * :* .***:** :.  :.:***:****:** **  *  :*

mNDST1          YFRDHNIELSKLLYKMG-QTLPTWLREDLQNTR---------------------------
mNDST2          FFRNHNLELSKLLSRLG-QPAPLWLREELQHSSVG-------------------------
Sfl             YYLNHNTALVKLLKKLGSRPIPQWLKDDLSTGTXXQLFGGGGGGGSVGSIGGTKYRKLRA
                :: :**  * *** ::* :. * **:::*.

mNDST1          ------------------------------------------------------
mNDST2          ------------------------------------------------------
Sfl             KLRKQLHWLHVAAENAESAQRRRRHNXQDENLTELRWKIYHTRFQFSLLASFCK
```

Figure S1. Alignment of mouse and *Drosophila* NDST amino acid sequences.
Amino acid sequences of mouse Ndst1 (mNDST1), Ndst2 (mNDST2), and *Drosophila* Sulfateless (Sfl) are aligned using Clustal W. Residues that are similar or identical between three sequences are marked with dots and asterisks, respectively. The N-deacetylase domains and the sulfotransferase domains are highlighted in yellow and green, respectively. The Tyr_{315} residue of the Sfl protein sequence, where T2A-mNdst cDNA fusion constructs are inserted in *Ndst1* and *Ndst2* KI alleles, is shown by underline. The insertion disrupts the *sfl* gene at the beginning of the deacetylase domain.

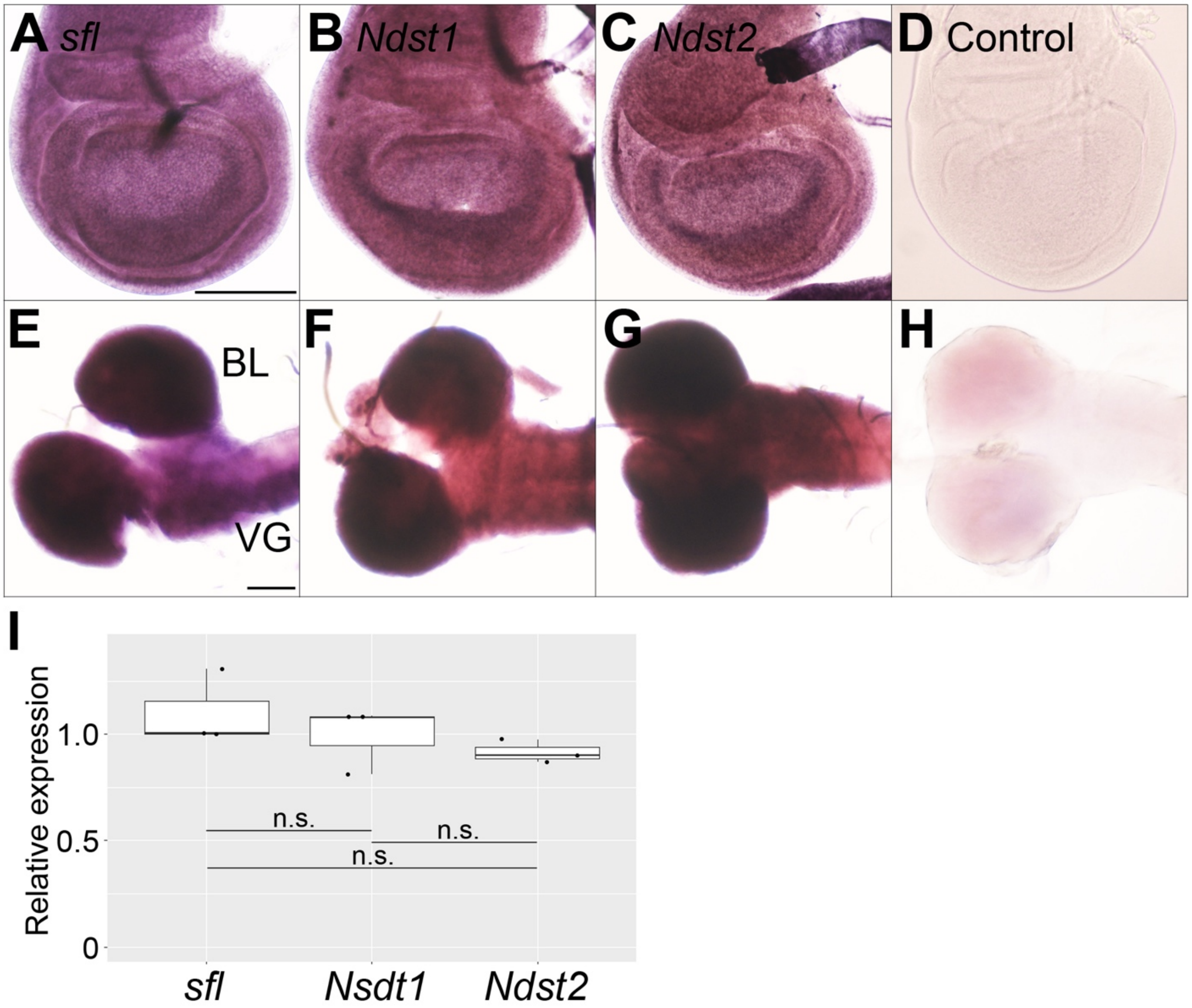

Figure S2. In situ RNA hybridization of *Ndst* mRNAs.
(A-H) In situ RNA hybridization showing expression of *sfl* (A and E), *Ndst1* (B and F), and *Ndst2* (C and G) mRNAs in the third instar larval wing disc (A-D) and CNS (E-H). The tissue samples were dissected from wild-type (A, E, D, and H), *Ndst1 KI* (B and F), and *Ndst2 KI* (C and G) animals. D and H show a control wild-type wing disc (D) and CNS (H) hybridized with a *sfl* sense probe. BL, brain lobe; VG, ventral ganglion. Scale bars, 100 µm. (I) RT-qPCR analysis of *sfl* in wild-type, *Ndst1* in *Ndst1* KI, and *Ndst2* in *Ndst2* KI. RNA samples were prepared from the whole third instar larvae (n=3 for each genotype). n.s., not significant (two-sided, unpaired t-test).

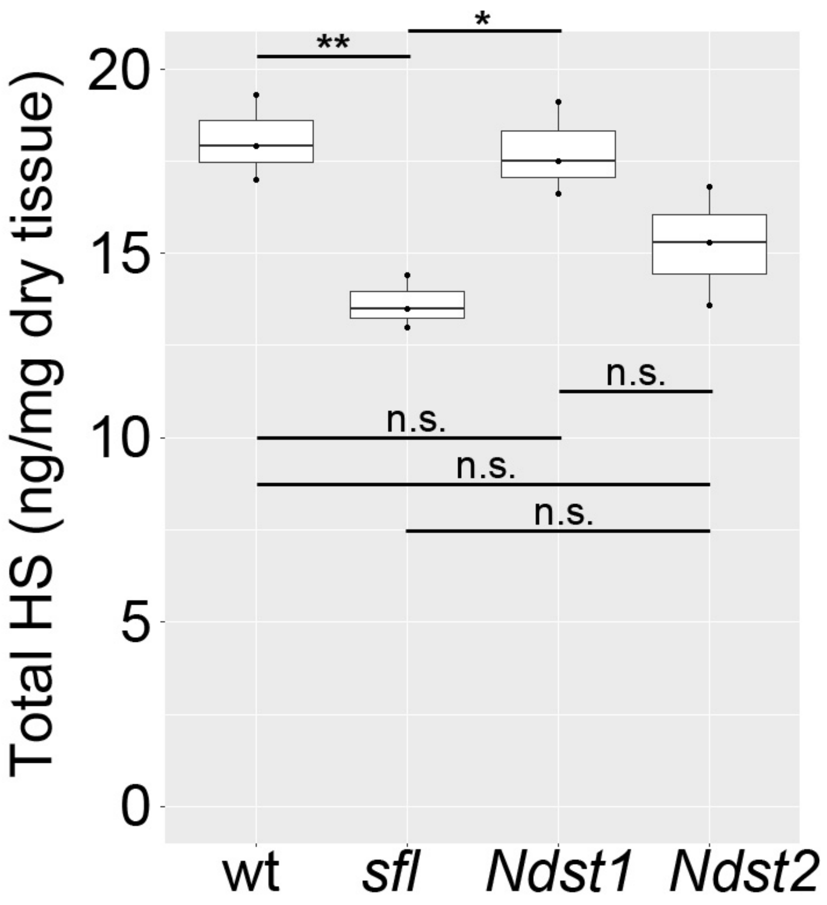

Figure S3. Total HS of *mNdst1* and *mNdst2* KI alleles.
Comparison of total HS (ng/mg dry tissue) from wild-type (wt), *sfl*, *Ndst1*, and *Ndst2* recovered in disaccharide analyses. No significant difference in the amount of HS from wild-type (wt) and the two *Ndst* KI alleles was detected.
